# Retraction Note: Hybrid nanoparticulate system of Fluvastatin loaded phospholipid, alpha lipoic acid and melittin for the management of colon cancer

**DOI:** 10.1038/s41598-024-78897-z

**Published:** 2024-11-13

**Authors:** Mohamed A. Alfaleh, Omar Fahmy, Mohammed W. Al-Rabia, Mohammed A. S. Abourehab, Osama A. A. Ahmed, Usama A. Fahmy, Helal H. Alsulimani, Shaimaa M. Badr-Eldin, Hibah M. Aldawsari, Bander M. Aldhabi, Awaad S. Alharbi, Nabil A. Alhakamy

**Affiliations:** 1 Department of Pharmaceutics, Faculty of Pharmacy, King Abdulaziz University, Jeddah, Saudi Arabia; 2 Vaccines and Immunotherapy Unit, King Fahd Medical Research Center, King Abdulaziz University, Jeddah, Saudi Arabia; 3 Department of Urology, Universiti Putra Malaysia, 43400 Selangor, Malaysia; 4 Department of Medical Microbiology and Parasitology, Faculty of Medicine, King Abdulaziz University, 21589 Jeddah, Saudi Arabia; 5 Department of Pharmaceutics, and Industrial Pharmacy, Faculty, of Pharmacy, Minia University, Minia, Egypt; 6 Department of Pharmaceutics, Faculty of Pharmacy, Umm Al-Qura University, Makkah, Saudi Arabia; 7 Department of Pharmaceutics and Industrial Pharmacy, Cairo University, Cairo, 11562 Egypt; 8 Center of Excellence for Drug Research and Pharmaceutical Industries, King Abdulaziz University, 21589 Jeddah, Saudi Arabia; 9 Mohamed Saeed Tamer Chair for Pharmaceutical Industries, King Abdulaziz University, 21589 Jeddah, Saudi Arabia

Retraction of: *Scientific Reports* 10.1038/s41598-022-24151-3, published online 14 November 2022

The Editors have retracted this Article. After publication, concerns were raised regarding irregularities in the flow cytometry plots presented in Fig. 7. The Authors have explained these irregularities, but have been unable to share the underlying raw data upon request.

Further checks by the Publisher found further potential irregularities in the data presented in Figs. 10 and 11. The Authors have stated that this and other cell line experiments in the study (Figs. 5–11) were performed by a third party, and the raw data are not available.

The Editors therefore no longer have confidence in the presented data.

The Publisher has not been able to obtain current email addresses for Mohamed A. Alfaleh and Usama A. Fahmy. None of the other Authors have responded to any correspondence from the Publisher about this retraction.

